# The Tuning in to Kids parenting program delivered online improves emotion socialization and child behavior in a first randomized controlled trial

**DOI:** 10.1038/s41598-024-55689-z

**Published:** 2024-02-29

**Authors:** Susan C. A. Burkhardt, Patrizia Röösli, Xenia Müller

**Affiliations:** Institute for Educational Support for Behavior, Social-Emotional, and Psychomotor Development, University of Teacher Education in Special Needs, Zurich, Switzerland

**Keywords:** Psychology, Randomized controlled trials

## Abstract

Emotion-focused parenting interventions have only rarely been evaluated systematically in Europe. This study investigates the effectiveness of “Tuning in to Kids” (TIK) from Australia delivered online in a randomized controlled trial. TIK is a six-week emotion-focused group parenting program that has shown to improve many aspects of parent emotion socialization as well as child problem behavior in several different countries across cultures. Parents (N = 141) of children between 3 and 6 years of age were included in the study and randomly assigned to an intervention and wait-list control group. The intervention was delivered online due to the worldwide COVID-19 pandemic in spring 2021 (intervention group) and one year later (control group) in Switzerland. Parents’ beliefs about emotions, their reported reactions to the child’s negative emotions, family emotional climate, and child behavior (internalizing and externalizing) improved after the intervention and stayed better until the 6 months follow-up in the intervention group, but not in the wait-list controls. Adherence to the program was very high. This study shows that parent emotion socialization practice is changeable with small effects even on child behavior and even after online delivery. This possibly makes Tuning in to Kids a promising emotion-focused parenting intervention when delivered online as an interactive group webinar.

## Introduction

Early childhood, also known as the emotion socialization period, is a critical period for the development of socio-emotional skills^1^. Growing evidence shows that emotional competence is related to academic success, psychosocial competence, and well-being^2,3^. Emotional competencies include noticing and naming emotions in self and others, understanding how emotions occur, and what possible consequences of these emotions are. In regulating emotions, children use cognitive, physical, and behavioral strategies to alter their emotional experience or expression (ibid.).

Whereas factors within the child’s person – such as cognitive flexibility, and temperament—determine his emotional competence and interactions with his parents to a certain degree, experiences within the family, classroom, and peer group also contribute to the emotional development^4^.

### Emotion socialization within the family

Emotion socialization – that means learning about emotions, emotion expression and emotion regulation – happens in every social interaction^5^ but for very young children this occurs mainly in the family context. Parents influence their children’s emotional competence and skills in many, also in unconscious, ways (ibid.). Most of the emotion-related knowledge is not explicitly taught but experienced and role-modeled and thus learned implicitly^6^. Emotion socialization that impacts the development of emotion regulation is characterized mainly by three components: (1) observational learning or modeling, (2) parenting practices related to emotions and emotional expression, and (3) emotional family climate. The latter includes parenting style, attachment, marital relationship quality, family expressiveness, and parental emotion regulation skills^7^. Especially emotion regulation is a key element of social competence. Emotion regulation is described as a complex interplay of competencies and processes that involve awareness, evaluation, maintenance, and modulation of emotional states to accomplish one's goals (ibid.). It’s a complex set of processes, including physiological, social, and cognitive components that may work consciously and deliberately, or unconsciously and automatically; they can be supported externally by a caregiver or self-managed. Emotion regulation therefore is seen as an ongoing task from womb to tomb which changes throughout life and is sensitive to learning and experiences.

In the last years, a growing body of studies has emphasized the importance of the parents’ emotion regulation as a prerequisite of their reaction to the child’s emotion and emotional expression and thus, for any parenting behavior^7^. This is why negative or insufficient parental emotion regulation seems to be related to poor executive functioning in preschoolers^8^. Whereas observational learning and modeling as well as the emotional family climate seem to be a rather indirect mechanism in emotion socialization, parenting practices related to emotions and emotional expression are a very direct socialization strategy where parents can more purposely interact with the child according to the child’s emotion or emotional expression. The way parents react to their child’s emotions is crucial for their emotional development. While the child’s temperament influences the relation between parental responses, and the child’s regulation strategies^9^, parents are herein also led by their own thoughts and feelings about certain emotions depending on their own emotional experience^6^. These so-called meta-emotions^6^ are sometimes also shaped culturally. When parents for example believe that the expression of anger is a sign of low self-control and that self-control is important, they possibly criticize children when they show anger or even punish them. By this, the child learns that anger must not be shown or not even felt, and parents miss the opportunity to teach their child about anger and how anger can be regulated or expressed in a socially accepted way in the respective cultural context. Gottman and DeClaire^10^ observed family interactions and found four different styles of parent emotion-related socialization behaviors: (1) emotion dismissing, (2) emotion disapproving, (3) laissez-faire, and finally (4) emotion coaching. The first three styles pay less attention to the emotion itself, but the fourth style emotion coaching does (ibid.)^11^. When dismissing emotions, parents can be warm and supportive in that they want their children to feel “better” (e.g. encouraging children to do something fun when they are sad), but they miss the emotional root of the behavior. By this, the child does not learn emotional vocabulary and no emotion regulation skills other than distraction or cheering up. Parents who criticize or even punish their children for their emotions or emotional expressions follow the emotion disapproving parenting style. On the contrary, when parents encourage emotional expression but don’t help and guide children to regulate or problem-solve, their style is called laissez-faire. All these three styles, other than emotion coaching, lead to children who show poorer emotional competence^10^.

### Emotion Coaching

Emotion coaching parents validate the child’s emotions and at the same time help either to regulate the emotion or to solve the problem. Emotion coaching includes five steps: (a) being aware of the child’s emotion, especially if it is still at a lower intensity; (b) view the child’s emotion as an opportunity for intimacy and guidance; (c) to listen empathetically, and to accept and validate children’s feelings and emotions (d) help the child to use words to describe how they feel and express their emotions; and (e) if necessary, assist them with problem-solving (while setting limits)^6^. Emotion coaching is described as an important technique for sustainable emotional and behavioral well-being: It has a positive influence on physiological and neurobiological development because it helps to establish a good vagal tone and gives children a skill base to engage in resilient and prosocial behaviors (ibid.). Children feel listened to and taught through empathic support, role modeling, and co-constructed problem-solving. Emotion coaching allows adults to remain calmer even when they must deal with intense child emotions, thereby optimizing access to behavior control and rationality. Parents are “allowed” and even must stay in an observing, a coaching role, and should not go to deep into their child’s emotion themselves, even though especially strong emotions can be contagious. Especially when a child is angry and misbehaves, it often happens that the parent eventually gets angry himself instead of staying calm and in a coaching role. When parents succeed to do so, they feel more confident in their interaction with children, which again makes them calmer and supports their own emotion regulation^7^. Emotion coaching even enhanced the therapeutic effects of psychotherapy in children with oppositional defiant disorder^12^.

Because child behavior problems are challenging for parents and the child himself^13^, promising ways of prevention and (early) intervention also need to be implemented in Switzerland. After many years of mainly behavioral approaches in parenting programs^14^, we face, like in psychotherapy, strategies that emphasize the emotion underlying a specific behavior^10,15^. Emotion-focused approaches to parenting aim to (1) promote emotion regulation skills for parents, (2) enhance the emotional climate of the family, (3) consider parental meta-emotion philosophy and (4) promote emotion-related socialization behaviors^3^. This combination of skills in turn assists children to understand and regulate their emotional experience, which, as a consequence, helps them to adjust to the situation better. Further, it builds neurocognitive and physiological flexibility in childhood, an emerging marker of child adjustment^16^. Especially when parents want to improve the attachment relationship with their child or when parents and children alike experience emotion dysregulation, an emotion-focused intervention seems promising^17^.

### The Tuning in to Kids parenting program

Whereas many traditional parenting programs aim to improve parents’ attitudes and parenting styles in general^14^, they miss the point of discussing and teaching specific emotion-related parenting techniques, that are important to model and help the child develop emotional competence. Emotion-focused parenting programs close this gap. Tuning in to Kids (TIK)^®^^18^ from Australia is among the best-documented^19^ and evaluated programs; it shows medium to large effect sizes^9,20^ in this category of emotion-focused parenting programs. Because Australia and Switzerland are similar with regard to emotion regulation, i.e. reappraisal^21^, TIK could be an interesting program for the Swiss population. TIK is a six-session group parenting program developed based on the theories of emotion socialization^22^. Additionally, it contains psychoeducation elements about the developmental stage of the children. Parents learn skills for their own emotion awareness and regulation, explore their meta-emotion philosophy, and learn the basis of the emotion coaching parenting style, meaning that parents may be permissive when it comes to emotions but should be strict when it comes to (mis-) behavior – so all feelings and desires are allowed but not every behavior is acceptable (Ginott, 1965, c. f.^10^). This approach follows the mindful parenting concept where the child is not judged for their feelings but behaviors may be commented on or the parent sets limits^10^. It targets parents with children aged 3–10 and aims to improve parental awareness of emotions, emotional regulation, and well-being to enhance the family emotional climate, and to strengthen parents’ positive attitudes and beliefs about parenting as well as their skills in emotion coaching; thus, to assist children to develop their emotional competence. TIK was originally designed as a universal intervention program^23^ but was already adapted for traumatized families^24^, fathers^25^, and other age groups like teenagers^26^ and toddlers^27^. In all these TIK programs, parents are taught the five consecutive steps of emotion coaching in different exercises, with content specified as core, optional, or home activities (e.g. practicing an emotion talk exercise with their children, practicing the five steps of emotion coaching, using an emotion diary, and completing other emotion-related activities involving their child). They consider how their family of origin experiences might have shaped their parenting and try the emotion coaching approach. Activities include psychoeducation, role-play examples of emotion dismissing versus emotion coaching parenting, use of handout materials, practice exercises, group discussion, and activities for practicing at home. Thereby, emphasis was put on parents becoming aware of their own and their children’s emotions, including physiological symptoms, with a focus on understanding the function of children’s behavior and emotions. Furthermore, parents learned skills in regulating their own emotions, especially managing their anger. By this, TIK focuses on parental emotion socialization practices with the expectation that improving these will lead to improvements in children’s emotional competence and also their behavior^28^.

The six sessions of the program cover the following themes: What is emotional competence and why is it important; the five steps of emotion-coaching, noticing emotions at a low intensity (session 1), emotional awareness, naming emotions, tuning in to emotions, meta-emotion philosophy, emotional parenting styles (session 2), mindfulness, developing empathy, emotional vocabulary (session 3), mindfulness, self-care, reacting to fear and anxiety, problem-solving (session 4), understanding own and children’s anger, reacting to anger, setting limits to angry behavior, dealing with siblings fighting (session 5), repetition of themes or time for postponed exercises (session 6).

In the last years, research has shown the effectiveness of TIK in several settings: whether delivered to parents of children with no severe problems, with internalizing or externalizing behavior problems^29^, children’s behavior improved as well as their emotion regulation competencies^30^, the family emotional climate calmed down and parents also improved their emotion regulation skills^18^ at post-intervention and also at a six months follow-up. These results were replicated in many different countries with different cultures and traditions of emotional expression^22^.

In recent years, the number of online parenting programs has increased, in part due to the COVID-19 pandemic and following lockdowns. The literature suggests that the advantage of online delivery is that a larger number of parents can be reached with greater cost-effectiveness than face-to-face delivery. Although there is still a lack of studies examining the effectiveness of these programs, there is a body of research that concludes that self-guided online support programs reduce child behavioral and emotional problems and improve parental mental health^31,32^. Also, there is only limited research on the effectiveness of interactive group trainings that are delivered online with a professional group facilitator. TIK as an emotion-focused parenting program has, to our knowledge, not been evaluated as a live interactive group webinar with a closed group of parents and a group leader yet. An advantage of an interactive group webinar is the geographical flexibility for the participants while direct interactions with other group members and the group leader are possible; the effects of webinars seem to be greater compared to asynchronous online delivery^33^. TIK participants are taught various emotional skills, including reflecting on their own emotions and emotion regulation skills. It is not clear if improvement in emotional competencies can be achieved after a TIK webinar.

This study aimed to apply an emotion socialization parenting program for the first time as a webinar and evaluate its effectiveness in a randomized controlled trial thus, test, if TIK is an appropriate emotion-focused parenting program when delivered as an interactive group webinar.

In accordance with previous research on the TIK program in other countries and cultures, we hypothesize that the TIK training would even when delivered online.

1. Enhance parent emotion-related socialization behaviors,
2. Change attitudes toward emotions in parents,
3. Calm the family’s emotional climate, and as a secondary outcome:
4. Improve child behavior.

## Results

### Analyses

All statistical analyses were performed using the SPSS software, version 28.0.1.0.

#### Preliminary analyses

Explorative data analyses showed that the assumptions of normal distribution were not violated. Pearson’s *χ*^2^- and *t* tests were calculated, to identify potential differences in demographic variables between groups. Further preliminary analyses were performed to check whether the assumptions of ANOVA were met, including normality of residuals, non-presence of extreme outliers, and homogeneity of variance.

Missing data were mostly due to item nonresponse and attrition problems, with missingness in the dependent variables ranging from 15% at pre-intervention to 43% at 6 months follow-up. Despite Little’s MCAR test^34^ was not significant and thus we assume missing values were completely at random, multiple imputations were run following best practice for handling moderate to large amounts of missing data. Multiple imputations in contrast to other methods estimating missing values seem to better depict the missing data, whereas other methods generate data that are more like the existing, not missing data^35^. We ran the SPSS multiple imputation procedure and generated five automatic imputations with no constraints. Data aggregation/pooling was not considered necessary since the five imputed data sets were identical. For the analyses described in the following, we used the imputed data.

#### Main analyses

To evaluate training effects, repeated measures analyses of variance were computed with the factors group (intervention group (IG) vs. control group (CG)) and time (pre, post, follow-up), and effect sizes (partial η^2^) were calculated. Preliminary analyses had shown that the child’s gender was correlated with all dependent variables, it therefore was included as a covariate. According to Cohen^36^, we consider effect sizes as small (η^2^_p_ = 0.01), medium (η^2^_p_ = 0.06), and large (η^2^_p_ = 0.14). Because all hypotheses were directed, we calculated the single-tailed *p*-values for the significance of the findings and the Bonferroni-Holm method for correcting potential alpha cumulation. In almost all dependent variables significant time effects were present: their values dropped in both groups between pre- and post-intervention (Tables 1, 2, 3). Moreover, significant time x group interaction effects were found, indicating effects of the intervention.

**Table 1:** Means, standard deviation, internal consistency, and effect sizes for parent outcomes*.*

	*M* (*SD*)						*F*	η^2^	Internal consistency		
	T0	T1			T2						
	IG (N = 66)	CG (N = 59)	IG (N = 66)	CG (N = 59)	IG (N = 66)	CG (N = 59)	Time × group		T0	T1	T2
Emotional beliefs (MESQ)											
Emotion dismissing	2.91 (1.14)	3.07 (1.10)	1.80 (1.40)	2.38 (1.55)	1.46 (1.45)	2.24 (1.63)	2.70*	0.03	0.96	0.98	0.98
Emotion disapproving	2.74 (1.12)	2.63 (1.07)	1.66 (1.32)	2.09 (1.42)	1.39 (1.38)	1.88 (1.42)	3.58*	0.03	0.95	0.96	0.96
Laissez-Faire	3.81 (1.33)	3.86 (1.35)	2.77 (2.08)	3.04 (1.95)	2.22 (2.12)	2.92 (2.10)	1.40	0.01	0.92	0.94	0.96
Emotion coaching	5.12 (1.65)	5.08 (1.72)	3.98 (2.76)	4.08 (2.50)	3.16 (2.95)	3.80 (2.71)	0.92	0.01	0.99	0.99	0.99
Coping with children’s negative emotions (CCNES)											
Punitive reactions	1.61 (0.69)	1.62 (0.52)	0.99 (0.75)	1.25 (0.93)	0.85 (0.85)	1.13 (0.89)	1.80^+^	0.02	0.92	0.95	0.96
Parental distress reactions	2.34 (0.97)	2.31 (0.88)	1.46 (1.22)	1.81 (1.30)	1.33 (1.35)	1.65 (1.28)	1.87^+^	0.02	0.88	0.91	0.92
Emotion-focused reactions	4.86 (1.58)	5.52 (1.55)	2.99 (2.21)	3.99 (2.55)	2.48 (2.38)	3.76 (2.71)	1.68^+^	0.01	0.97	0.98	0.98
Problem-focused reactions	5.69 (1.65)	5.51 (1.65)	4.21 (2.95)	4.41 (2.76)	3.43 (3.10)	4.10 (2.90)	0.37	0.00	0.98	0.99	0.99
Minimization reactions	1.91 (0.93)	1.94 (0.95)	1.10 (0.88)	1.45 (1.07)	0.95 (0.99)	1.26 (1.01)	1.53	0.01	0.90	0.92	0.93
Expressive encouragement	5.11 (1.71)	5.17 (1.68)	4.22 (2.97)	4.13 (2.69)	3.34 (3.05)	3.79 (2.77)	0.34	0.00	0.97	0.99	0.99

Effects of time × group; *F*(1, 124); ^+^*p* < 0.10, *p < 0 05; all hypotheses were tested one-tailed, and gender of the child was included as a covariate.

*IG* intervention group, *CG* control group.

**Table 2:** Means, standard deviation, internal consistency, and effect sizes for emotional family climate.

	*M* (*SD*)						*F*	η^2^	Internal consistency		
	T0		T1		T2						
	IG (N = 66)	CG (N = 59)	IG (N = 66)	CG (N = 59)	IG (N = 66)	CG (N = 59)	Time × group		T0	T1	T2
Emotional family climate											
Inconsistent parenting (APQ)	2.58 (0.74)	2.49 (0.86)	1.70 (1.26)	1.98 (1.31)	1.45 (1.37)	1.85 (1.37)	2.28**	0.02	0.93	0.96	0.97
Harsh discipline (APQ)	3.01 (0.87)	2.88 (1.02)	1.99 (1.47)	2.20 (1.46)	1.67 (1.58)	1.97 (1.45)	1.88**	0.02	0.95	0.98	0.98
Attachment (EBI)	2.98 (1.02)	2.98 (1.06)	2.30 (1.66)	2.40 (1.60)	1.95 (1.74)	2.26 (1.67)	0.25	0.00	0.91	0.95	0.96
Spouse relationship (EBI)	2.41 (0.87)	2.33 (1.10)	1.68 (1.23)	1.85 (1.41)	1.39 (1.40)	1.75 (1.46)	1.21	0.01	0.86	0.90	0.94

Effects of time × group; *F*(1, 124); **p < 0.01; all hypotheses were tested one-tailed, and gender of the child was included as a covariate.

*IG* intervention group*, CG* control group.

**Table 3:** Means, standard deviation, internal consistency and effect sizes for child outcomes.

Behavior problems (SDQ)	*M* (*SD*)						*F*	η^2^	Internal consistency		
	T0		T1		T2						
	IG (N = 66)	CG (N = 59)	IG (N = 66)	CG (N = 59)	IG (N = 66)	CG (N = 59)	Time × group		T0	T1	T2
Internalizing problems	4.63 (3.64)	3.61 (2.35)	2.29 (2.81)	3.00 (3.02)	2.53 (3.17)	2.42 (3.10)	4.56**	0.04	0.75	0.81	0.85
Externalizing problems	7.09 (3.52)	6.05 (3.63)	4.41 (3.74)	4.80 (4.16)	3.68 (4.17)	4.86 (4.45)	5.00**	0.04	0.83	0.85	0.90
Global problem score	11.73 (5.53)	9.66 (5.04)	6.69 (5.49)	7.80 (6.72)	6.21 (6.72)	7.28 (6.95)	5.34**	0.04	0.85	0.88	0.92

Effects of time × group; *F*(1, 124); **p < 0.01; all hypotheses were tested one-tailed, and gender of the child was included as a covariate.

*IG* intervention group, *CG* control group.

### Parenting outcomes

#### Parent reported beliefs about children’s emotions and emotion socialization (MESQ)

**Figure 1:**
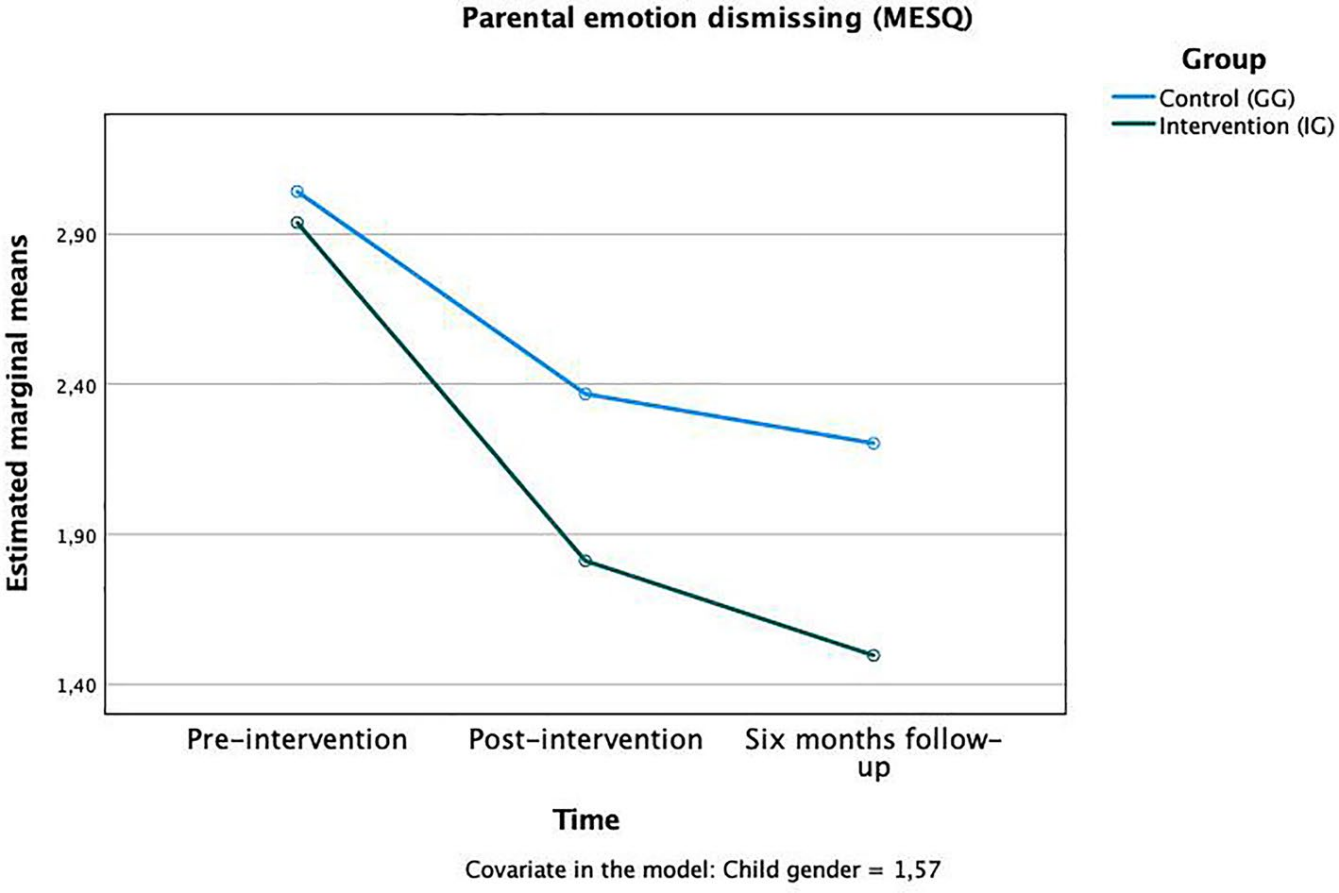
Time × group interaction in the “emotion dismissing” subscale of the MESQ.

**Figure 2:**
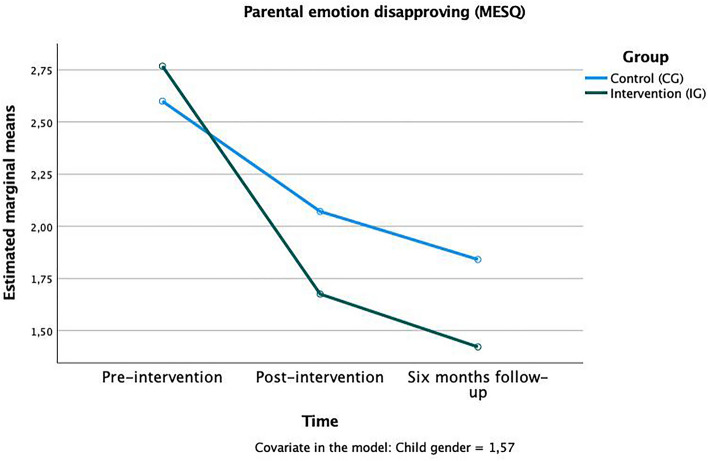
Time × group interaction in the “emotion disapproving” subscale of the MESQ.

For all MESQ subscales, there was a significant main effect for “time” present: “emotion dismissing” *F*(1,124) = 4.214, *p* < 0.05, η^2^_p_ = 0.07, „emotion disapproving “*F*(1,124) = 3.667, *p* < 0.05, η^2^_p_ = 0.06”, “laissez-faire” *F*(1,124) = 4.869, *p* < 0.01, η^2^_p_ = 0.07 and emotion coaching “*F*(1,124) = 5.611, *p* < 0.01, η^2^_p_ = 0.09”. For testing our hypotheses, the time × group interaction result is the most important: Significant time x group interactions were found in the two subscales “emotion dismissing” *F*(1,124) = 2.70, *p* < 0.05, η^2^_p_ = 0.02 (Fig. 1) and “emotion disapproving” *F*(1,124) = 3.58, *p* < 0.05, η^2^_p_ = 0.03 (Fig. 2). Both scales capture rather negative (neglecting/emotion dismissing) or even punitive, harsh (emotion disapproving) reactions to children’s emotions. Parents of the IG had lower values at both measurement points after the intervention compared to the CG, indicating fewer negative reactions to children’s emotions. The effects occurred already between pre- (T0) and post-intervention (T1) and continued to be significant until the 6-months follow-up (T2) in that way, that the group mean stayed stable in the CG and dropped further in the IG. No group differences were found in the “emotion coaching” and “laissez-faire” subscales (Table 1).

#### The coping with children’s negative emotions scale (CCNES)

Also for the CCNES subscales, significant main effects for “time” were found: “punitive reactions” *F*(1,124) = 4.212, *p* < 0.05, η^2^_p_ = 0.07, “parental distress” *F*(1,124) = 4.156, *p* < 0.05, η^2^_p_ = 0.06, “emotion-focused” *F*(1,124) = 6.612, *p* < 0.01, η^2^_p_ = 0.01, “problem-focused” *F*(1,124) = 6.290, *p* < 0.01, η^2^_p_ = 0.09, “minimization” *F*(1,124) = 6.377, *p* < 0.01, η^2^_p_ = 0.01, “expressive encouragement” *F*(1,124) = 6.334, *p* < 0.01, η^2^_p_ = 0.01.

**Figure 3:**
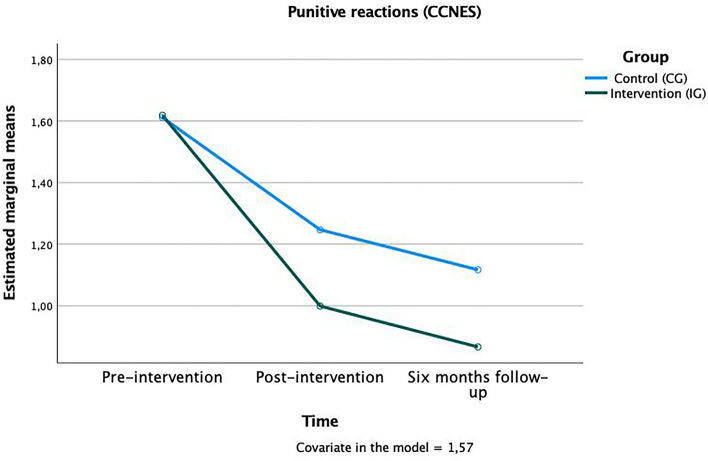
Time × group interaction in the “punitive reactions” subscale of the CCNES.

**Figure 4:**
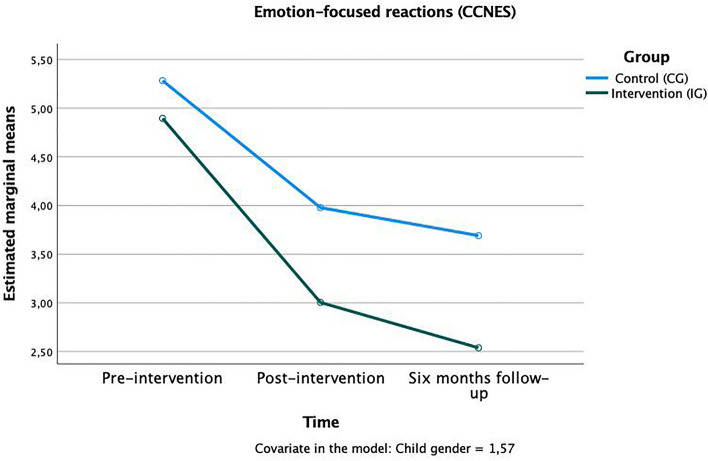
Time × group interaction in the “emotion-focused reactions” subscale of the CCNES.

**Figure 5:**
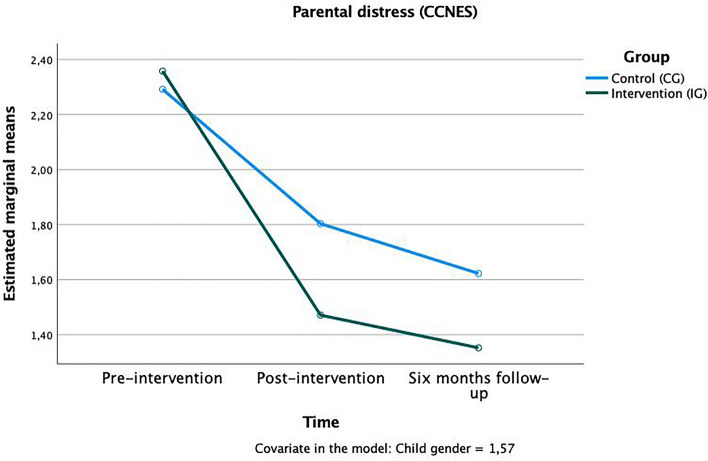
Time × group interaction in the “parental distress” subscale of the CCNES.

Regarding the parents ‘ reactions to the child’s negative emotions, trends were found in time x group interactions in three subscales that, again, rather describe harsh and negative reactions as well as the parent’s own emerging distress: “punitive reactions” *F*(1,124) = 1.80, *p* < 0.10, η^2^_p_ = 0.02 (Fig. 3); “emotion-focused reactions” *F*(1,124) = 1.68, *p* < 0.10, η^2^_p_ = 0.01 (Fig. 4), and “parental distress reactions” *F*(1,124) = 1.87, *p* < 0.10, η^2^_p_ = 0.02 (Fig. 5). In all these scales the mean value in the IG was lower after the training and continued to drop until the follow-up after six months, meaning that parents who attended the training used fewer negative strategies to cope with children’s emotions. The other subscales (minimization reactions, expressive encouragement, and problem-focused reactions) showed no significant time x group effects (see Table 1).

### Family outcomes

#### Alabama parenting questionnaire

Like the parenting scales described above, main effects for “time” were found in both subscales: “inconsistent parenting” *F*(1,124) = 5.053, *p* < 0.01, η^2^_p_ = 0.08 and “harsh discipline” *F*(1,124) = 7.798, *p* < 0.001, η^2^_p_ = 0.11.

**Figure 6:**
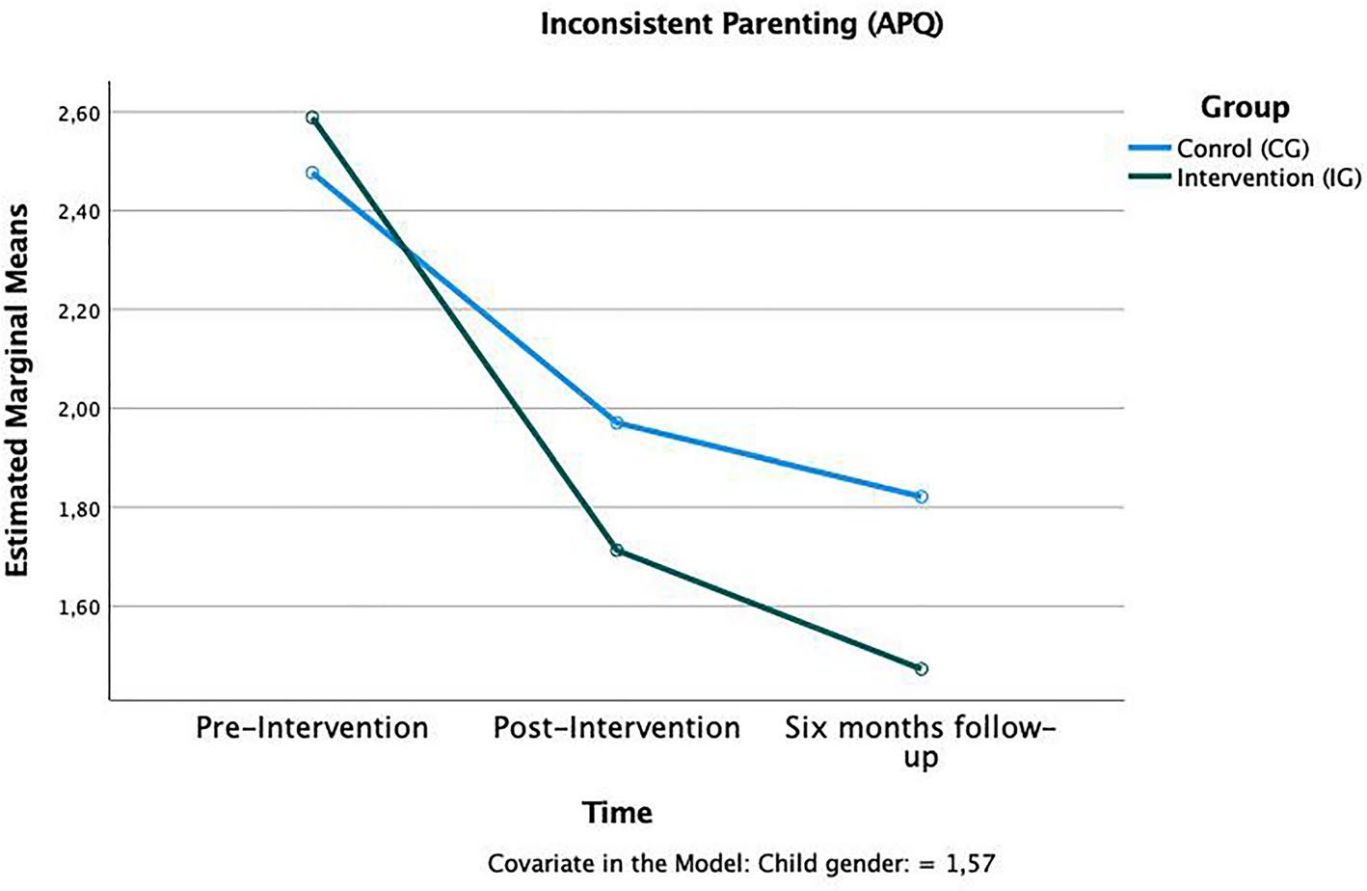
Time × group interaction in the “inconsistent parenting” subscale of the APQ.

**Figure 7:**
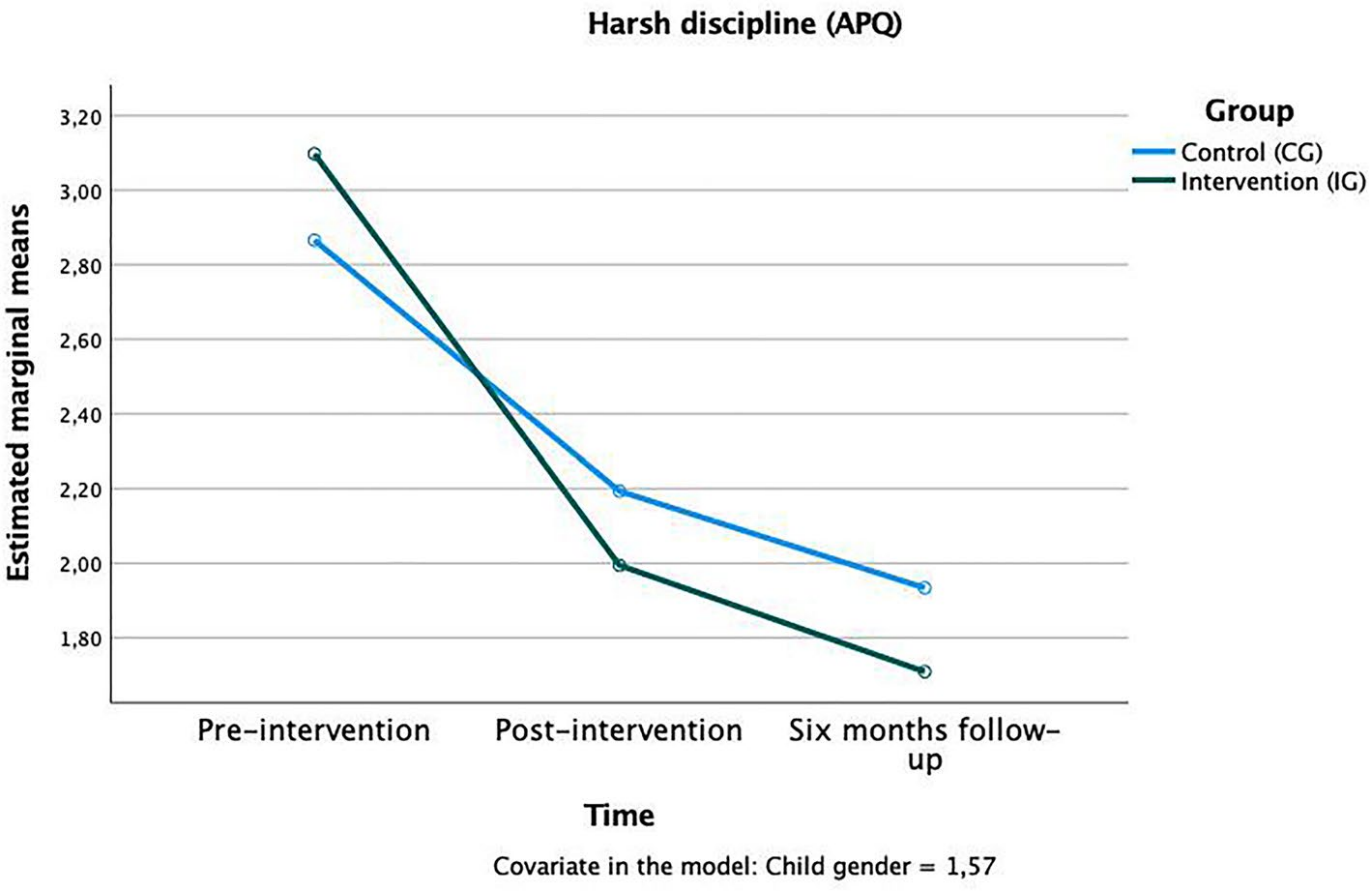
Time × group interaction in the “harsh discipline” subscale of the APQ.

Training effects were present on both subscales of the Alabama parenting questionnaire: “inconsistent parenting” *F*(1,124) = 2.28, *p* < 0.01, η^2^_p_ = 0.02 (Fig. 6) and harsh discipline *F*(1,124) = 1.88, *p* < 0.01, η^2^_p_ = 0.02 (Fig. 7). In both scales, parents of the IG reported less of the respective, rather negative, parenting preferences after the training was completed and at the follow-up.

#### Parental stress index

In the subscales of the parental stress index, there were also main effects for “time” present: “attachment” *F*(1,124) = 6.283, *p* < 0.01, η^2^_p_ = 0.09 and spouse relationship *F*(1,124) = 7.994, *p* =  < 0.001, η^2^_p_ = 0.12.

None of the EBI subscales (attachment or spouse relationship) showed significant time x group interactions (Table 2).

### Secondary outcomes—child behavior

Child behavioral adjustment, measured by the strength and difficulties questionnaire (SDQ^37^), such as internalizing and externalizing behavior problems was within the normal range, showing no concerning level of behavior problems in either group at any time. Time effects were present only in the externalizing problems subscale of the SDQ: internalizing problems *F*(1,124) = 1.723, *p* > 0.05, externalizing problems *F*(1,124) = 3.136, *p* < 0.05, η^2^_p_ = 0.05, global problem score *F*(1,124) = 2.943, *p* > 0.05.

Still, for internalizing behavior problems, there was a significant time x group interaction effect, sphericity assumed *F*(1,124) = 4.56, *p* < 0.01. The small effect (η^2^_p_ = 0.04,) occurred between pre- and post-intervention and stayed stable until the 6 months follow-up. Children of the IG had the most dramatic change in this measure. Similar effects were found for externalizing behavior problems: *F*(1,124) = 5.00, *p* < 0.01, η^2^_p_ = 0.04, and the SDQ global problem score *F*(1,124) = 5.34, *p* < 0.01, η^2^_p_ = 0.04. In these measures also the effect appeared mainly between pre- and post-intervention, but the IG continued to show reduced problem behavior until the follow-up at six months post-intervention, whereas the CG stayed stable in all scales (Table 3).

### Credibility of effects evaluation

We formally evaluated our findings using a formal instrument for assessing the credibility of effect modification analysis (ICEMAN^38^). For all time x group interaction effects, the credibility was moderate (parenting measures) to high (emotional family climate and child behavior outcomes).

## Discussion

Like in other countries^17^, behavioral parenting programs are the most common evidence-based parenting programs in Switzerland; this study was the first randomized controlled trial (RCT) using an emotion-focused parenting program delivered as an interactive group webinar in Switzerland. It evaluated the efficacy of the TIK program as a universal intervention with parents of preschoolers without clinical behavior problems. Moreover, it is the first study with the TIK group program delivered solely online as a webinar.

Australia and Switzerland have similar cultures regarding emotion and emotion regulation reappraisal^21^. The effectiveness of the TIK program from Australia could be replicated in almost all hypothesized measures also in the Swiss webinar version: Six months after the training, parents in the IG still showed improvements on targeted aspects of parenting, and their children had fewer behavior problems. This supports theory and previous research suggesting that a change in parenting is linked to a change in child behavior^39^; this study found effects in both domains; parents and children alike: In line with previous TIK evaluation studies^29,40^, emotion dismissing and emotion disapproving decreased in the intervention group and stayed lower than in the control group. However, in this study, emotion coaching did not increase^23^. TIK studies often find that emotion dismissing is where the greatest changes occur. Unlearning dismissiveness is the easiest part for parents to learn as they develop new insights. However, applying the skills of the five steps is harder – especially in emotional moments (ibid). Some parents in Switzerland stated in a feedback form used after the last session that the emotion coaching technique was very easy to understand but not easy to apply and that they would appreciate more training; they regretted that the program already ended after six sessions. Booster sessions could be an option here.

The same is true for the subscales of the CCNES, measuring the parent’s reactions to the child’s negative emotions: slightly less punitive and parental distress and emotion-focused reactions were found post-intervention and at the follow-up after six months in the intervention group, meaning a decrease in the rather “non-supportive” subscales but no significant improvements on the “supportive” subscales like problem-focused reactions or expressive encouragement. The subscale emotion-focused reactions, where a small intervention effect was found, contrary to its name, measures an emotionally dismissive way of parenting^30^. Dismissing emotions can be done in a very calming and warm way; parents want their children to feel better, so they try to distract them or cheer them up^6^ and thus can easily be falsely considered as the best way to respond to a child’s emotions or rated as being a supportive parenting strategy.

This pattern of effects in the parent socialization outcomes supports the parental meta-emotion philosophy theoretical framework: The parent’s beliefs and feelings about emotions are directly linked to parental (automatic) reactions to the child’s unpleasant emotions^41^, whereas building new emotion socialization supporting parenting skills would need more training in our sample.

Effect sizes in general were small and sometimes only showed a trend on the *p* < 0.10 level. The biggest effects were present in child behavior problems, both internalizing and externalizing and global problems. These effects might be subject to desirability or expectation bias; questionnaires about the children’s behavior were answered by their parents who could not be blinded regarding the experimental condition. Moreover, parents may have rated their children’s behavior as being less problematic because in the TIK training, they learned about the developmental context of children and what behavior might be just normal in the respective developmental stage. Third, the first step of emotion coaching according to Gottman et al.^10^ is to notice and to address emotions already at a low level of intensity. So, the child’s needs for connection and maybe emotional clarification are met already before more intense emotions would lead to problematic behavior. Nevertheless, when parents experience their children behaving less problematically, this already is relieving^42^ and is an important improvement for their daily family life.

The fact, that effects of this study were small, needs more reflection: Effect sizes have been found to be larger in other TIK evaluations in and outside Australia^43,44^ in samples with behavior problems where the training was delivered in a group setting meeting in presence. It is obvious that a strong, experienced group leader greatly contributes to the uptake of the program^45^; in this study, all group leaders facilitated a TIK group for the very first (IG) and second (CG) time so it can be hypothesized that with greater experience in delivering this intervention effect sizes might increase. A recent meta-analysis concluded that the effects of emotional parenting practices sometimes can’t be proven in children’s outcomes^46^; this could have affected effect sizes or even covered effects. Another reason for the small effects, compared to previous TIK evaluation studies in similar samples^40^, is that the TIK training in Switzerland was delivered online for the intervention group. Otterpohl et al.^40^ had found no and hardly significant effects in some scales (e.g. the EBI) in Germany; in the Swiss interactive group webinar trial these effects could not be detected at all, although the population of the trials and the culture are quite similar: In an online setting, emotions and the impact of role plays are potentially buffered: people cannot move around, approach or leave each other; group dynamics and empathy with fellow group members are less intense than in real groups who meet in person for six weeks and experience a certain feeling of group coherence^47^. Also, the length of the 2.5 h session could have been tiring online, even more so, when technical problems occurred, possibly impairing the quality of the training delivery, and thus minimizing effects.

Other than the German study^40^, which evaluated training effects on parenting and family emotional climate, this trial included measures on the child level, where the largest, but still rather small, effects were found.

Also, standard deviations in most measures increased over time, as is often found in TIK studies^29,30,48^, implying that not all parents in the intervention group have improved similarly or at all. Mediation or latent profile analyses with larger sample sizes to detect patterns and predictors of change and development over time adequately would be a necessary and interesting future task.

Regarding the quality of the parental training delivery and to assess participants’ own efforts and engagement (e.g. how much and intensively they practiced at home between the training sessions), future TIK studies should assess the fidelity of the training delivery more objectively, e. g., by using observation scales for training delivery (fidelity and quality) and monitoring sheets for the parents’ engagement.

Measures used in this study were solely questionnaires, dependent on the parents’ opinion which could be influenced by expectation bias. Future studies should also use testing^30^, observation^44^ or/and psychophysiological measures (ibid.) to increase the objectivity and external validity of these first findings. Replication of these first results in a larger sample is desirable.

The sample of this study was highly educated and rather homogenous regarding socio-economic status and nationality living in a family with two parents present. Information about race/ethnicity was not collected. Participants lived in five different regions of the German-speaking part of Switzerland and had to be quite fluent in German to sign up for the study, understand and follow the TIK training, and to answer the research questionnaires. This is often a problem in parenting research^49^ that some groups are excluded because of language problems. Moreover, the participants had to be familiar with electronic devices and have access to one to be able to follow the online training. Children who already received psycho- or pharmacotherapy for their behavior problems were not allowed in the study. Thus, the sample of this first TIK study in Switzerland was rather privileged and not diverse; this reduces the generalizability of the results to other populations. Importantly, parents liked the program, and the attendance rate was very high; all parents in the IG received the full treatment and in the waiting controls hardly any parent missed out. Parent satisfaction is a key factor in implementation^50^.

This study was conducted during the worldwide COVID-19 pandemic that resulted in many social restrictions in almost every country. We found a decrease in nearly all measures in both groups between pre- and post-intervention. This may seem surprising but can partially be explained by the COVID-19 pandemic and the resulting lockdown. Some studies on emotions during the COVID-19 lockdown found that the intensity of negative *and* positive feelings and even anxiety, depression, and emotion regulation difficulties was experienced higher and more intense compared to before the lockdown^51^. This was also true for parents^52^. Also, it cannot be ruled out that the general drop in both groups is due to regression toward the mean as it is also present in other TIK trials^29,30^.

In this study, all data collection points were within the lockdown, but between pre- and post-intervention social restrictions were lifted a bit in Switzerland – from May 31st, 2021 (one week before the post-intervention data collection started), public events of 100 people (inside a venue) or even 300 people (outdoors) were allowed again; also private events with 50 (inside) to 100 (outside) guests were possible and home-office was not mandatory anymore for Swiss employees^53^. This might have contributed to the decreased intensity in our measures in both groups between pre- and post-intervention when the lockdown was not that strict anymore at post-intervention.

This study suggests that parent emotion socialization practice is changeable with small effects also on child behavior when delivered online in a parenting group setting. Thus, Tuning in to Kids might be a promising emotion-focused parenting intervention also in Switzerland when delivered as an interactive group webinar.

## Methods

### Recruitment and randomization

TIK Switzerland was carried out in five different German-speaking regions / cantons. In each canton, we first contacted the respective educational department and then were referred to the local structures (or contacted them ourselves where the initial requests remained unanswered).

Parents were recruited in early 2021 through mailings to child daycare institutions, via their local kindergarten or primary school principals and teachers, newspaper alerts, and a community parent–child meeting center e-mail newsletter.

Parents of children aged 3 to 6 years, one parent of each family and always the same parent, were eligible for participation if this parent could attend the TIK group meetings regularly and was willing to fill out the research questionnaires before and after the intervention, and six months later for a follow-up. An exclusion criterion was if the child already received psycho-therapeutical or pharma-therapeutical treatment. In addition, fluency in German was required for the participating parents to answer the questionnaires. Participation in the study and the training was free of charge and parents were not paid for retention or data collection.

**Figure 8:**
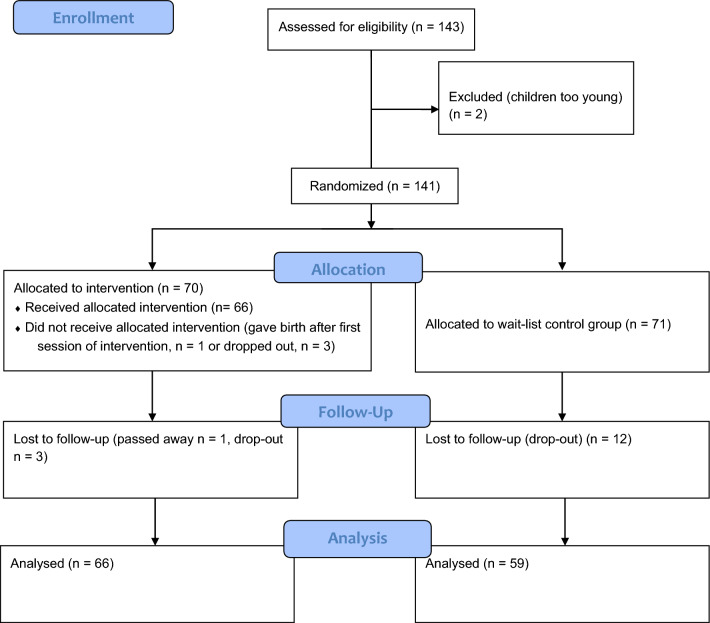
Participant flow.

N = 143 parents signed up for the study initially via an online form, at the same time giving their informed consent, and were assured that all information would be kept confidential and only would be used for research and organizational purposes within the TIK study. They could withdraw their consent and drop out of the study/sample at any time without explanation. Participants were then randomized into the intervention group (IG, N = 70, 50.4%) who would attend the TIK training from May to July 2021 in five groups and the wait list control group (CG, N = 71, 49.6%) who would attend it after the follow-up also in five groups, after two families had to be excluded because their children were too young (Fig. 8). Group trainings for the five wait CGs (one in each region) were offered between February and June 2022 after the completion of the follow-up; two of them could meet in person because of the regional provenience of the participants (Basel City and Münchenstein in Basel Country). The study protocol was approved by the head of research of the originating institution and the head of the department the three authors belong. All methods used were carried out in accordance with the ethical guidelines of the Swiss Psychologic Society, which are based on the American Psychological Association Ethics Code and the ethical guidelines of the German Psychological Society and the Professional Association of German Psychologists^54^.

### Sociodemographic information

Sociodemographic information was collected after randomization pre-intervention. Distribution of characteristics between the groups was balanced (see Table 4) with the two following exceptions: parent gender distribution was not equal between the two groups with more mothers in the CG and more fathers in the IG (*χ*^2^(1) = 12.81, *p* = 0.002, *Cramer-V* = 0.123, *p* = 0.002). In the CG n = 37 (62%) of the children were boys and n = 22 (37%) were girls. In the IG the gender ratio was more balanced with n = 32 (48%) girls and n = 34 (51%) boys; (*χ*^2^(1) = 12.81, *p* = 0.002, Cramer-*V* = 0.113, *p* = 0.002).

**Table 4:** Sample characteristics.

	IG (n = 70)	CG (n = 71)
	*M* (*SD*)/%	*M* (*SD*)/%
Family characteristics		
Marital status		
Unmarried	20%	20.3%
Married	69.2%	76.3%
Concubinage	6.2%	1.7%
Divorced	4.6%	1.7%
Partner is mother/father of the child	93.8%	96.6%
Siblings yes	77.3%	86.7%
Nationality		
Switzerland	51.5%	45.1%
Germany	25.7%	16.9%
Dual citizen	5.7%	8.4%
Other	11.3%	11.3%
Not specified	12.1%	18.3%
Parents		
Age of parents	39.15 (5.38)	39.28 (4.62)
Gender of parents		
Mother	85.7%^+^	90.1%^+^
Father	14.3%	8.5%
Not specified	0%	1.4%
Employment in %	87.7%	84.7%
Occupation in %	55.91 (30.501)	53.29 (29.80)
ISCO	2944.29 (1026.91)	739.73 (817.07)
Child		
Child gender		
Girl	48.5%*	37.3%*
Boy	51.5%	62.7%
Child age	4.99 (1.35)	4.91 (1.23)

*IG* intervention group, *CG* control group.

^+^*χ*^2^(1) = 12.81, *p* = 0.002, *χ^2^(1) = 12.81, *p* = 0.002.

### Intervention

The TIK training was offered for the intervention group (IG) in between April and June 2021 in five separate groups in five regions of the German-speaking part of Switzerland. Due to the ongoing COVID-19 pandemic and the respective restrictions in Switzerland, TIK training for the IG was delivered online. For the TIK group in Basel-City there were way more applications (N = 44) than we could offer participation in the Basel IG and Basel CG (max. 24 spots), so some Basel applications were included into other groups, that almost would have been too small at all (Solothurn and Thurgau). Because of online delivery this was a feasible way to include all interested parents by reorganizing them within the IG or CG no matter what their region of origin was. All five group leaders belong to the staff of the authors’ institution and were trained and certified as group leaders after a 2-day training by a certified TIK trainer from Germany in August 2020. All received group supervision throughout the intervention delivery (before and after session 1 and after session 3) to ensure they adhered to the structured manual and to address issues that arose during delivery. Regarding the dosage of the treatment, we can report that all parents included in the analysis received the full treatment, meaning they attended at least four sessions (out of six)^55^. Self-report fidelity checklists as provided in the TIK manual were completed by the group leaders after each session and 100% of core content was delivered.

### Data collection and measures

All participants filled out questionnaires delivered online four weeks before the IG had their first TIK training session (pre-intervention, before May 2021), two weeks after the last of these six sessions (post-intervention, June 2021) and six months thereafter (follow-up, January 2022). Confidentiality was confirmed and data was anonymized. For this, parents created their own four-digit code consisting of letters (“The first letter of my father’s/mother’s first name”) combined with numbers (“The month my father/mother was born, January would be 01”), e.g. “C5M12”, so data could not be linked with the parents’ or children’s names by the research team but still be matched between data collection points. To confirm that the parent always rated the same child’s behavior, if they had more than one child who would meet the inclusion criterion of being between 3 and 6 years of age, parents were asked at each data collection point to give the initials of the child. Initials were checked for concordance between the measurements.

#### Parenting measures

##### Parent reported beliefs about children’s emotions and emotion socialization

The *maternal emotional style questionnaire* (MESQ^10^ consists of 81 items on four subscales concerning the four different parenting styles and is answered on a 7-point Likert scale from “disagree” to “completely agree”. Parents rate how they cope with their child’s emotions of sadness, worry, fear, and anger. An example of emotion coaching (EC) is “When my child is angry, I take some time to try to experience this feeling with him/her”, emotion dismissing (ED) “I help my child get over sadness so he/she can move onto other things”, laissez-faire (LF) “When my child is angry, I don’t know what he/she expects from me” and emotion disapproving (ES) “When you let your child be angry, he/she thinks he/she will always get what he/she wants”. This questionnaire was extended by seven items, the parental emotional style questionnaire (PESQ)^56^, covering fear and worries (e.g. “when my child is worried, I want to know what he/she is thinking”; “I try to change my child’s worried moods into cheerful ones”). Four subscales were calculated with higher scores indicating higher agreement with the respective parenting style. Internal consistencies of the four subscales were good to excellent at all three measurements. Pre-intervention α_*ec*_ = 0.99, α_*ed*_ = 0.96, α_*lf*_ = 0.92, α_*es*_ = 0.95; post-intervention α_*ec*_ = 0.99, α_*ed*_ = 0.98, α_*lf*_ = 0.94, α_*es*_ = 0.96; and follow-up α_*ec*_ = 0.99, α_*ed*_ = 0.98, α_*lf*_ = 0.96, α_*es*_ = 0.96 (see Table 1).

##### Parental emotion socialization

The *coping with children’s negative emotions scale* (CCNES^57^) was used to measure parents’ emotion socialization practices. Parents used a 7-point Likert scale (1 = *very unlikely* to 7 = *very likely*) to agree or disagree. The CCNES consists of 12 scenarios of children experiencing negative emotions, e.g. “If my child loses some prized possession and reacts with tears, I would…”. Each scenario is followed by one item each of six subscales: punitive parental reaction (e.g. “tell him/her that's what happens when you’re not careful”), expressive encouragement (e.g*.* “tell him/her it's ok to cry when you feel unhappy”), emotion-focused reaction (e.g. “distract my child by talking about happy things”), problem-focused reaction (e.g. “help my child think of places he/she hasn't looked yet”), minimization (e.g. “tell my child that he/she is overreacting”), and parental distress (e.g. “get upset with him/her for being so careless and then crying about it”). The subscales showed excellent internal consistency in the current sample at all measurement points of measurement: Pre-intervention α_*punitive*_ = 0.92, α_*expressive*_ = 0.97, α_*emotion-focused*_ = 0.97, α_*problem-focused*_ = 0.98, α_*minimization*_ = 0.90, α_*parental distress*_ = 0.88; post-intervention α_*punitive*_ = 0.95, α_*expressive*_ = 0.99, α_*emotion-focused*_ = 0.98, α_*problem-focused*_ = 0.99, α_*minimization*_ = 0.92, α_*parental distress*_ = 0.91; and follow-up α_*punitive*_ = 0.96, α_*expressive*_ = 0.97, α_*emotion-focused*_ = 0.98, α_*problem-focused*_ = 0.99, α_*minimization*_ = 0.93, α_*parental distress*_ = 0.92.

#### Family emotional climate

In concordance with a previous evaluation of the TIK parenting program in Germany^40^, we used two scales of the Alabama Parenting Questionnaire (APQ)^58^, to measure general parenting tendencies. The subscale inconsistent parenting comprises five items, e.g. “you let your child out of a punishment early (like lift restrictions earlier than you originally said)” and showed excellent internal consistency: α_*pre*_ = 0.93; α_*post*_ = 0.96; α_*follow-up*_ = 0.97. The subscale “harsh discipline” consists of six items, e. g. “When your child starts negotiating with you, put your foot down.” Internal consistency was α_*pre*_ = 0.95; α_*post*_ = 0.98; α_*follow-up*_ = 0.98. Parents rated the frequency of their respective reaction on a 5-point scale from “never = 1” to “always = 5”.

Additionally, we used the attachment and the spouse relationship subscale of the German version of the parental stress index^59^, the Eltern-Belastungs-Inventar (EBI)^60^. Examples for the attachment scale (four items) are “In some situations, I wish I could better understand what my child is thinking and feeling.”

An example item for the spouse relationship subscale is “Since the child was born, my partner and I don't spend as much time together as I would like “. Both scales had a 5-point scale of agreement from “1 = not at all true” to “5 = totally true”. Internal consistencies were as follows: attachment: α_*pre*_ = 0.91; α_*post*_ = 0.95; α_*follow-up*_ = 0.96; spouse relationship: α_*pre*_ = 0.86; α_*post*_ = 0.90; α_*follow-up*_ = 0.94.

#### Secondary outcomes—child behavior

##### Behavioral adjustment

The *strengths and difficulties questionnaire* (SDQ^37^; was used to assess children’s behavioral adjustment. Parents rated their child using a 3-point scale (0 = *not true*, 1 = *somewhat tru*e, 2 = *certainly true*) with the 25 items on five subscales: conduct problems, hyperactivity/inattention, emotion problems, peer relationship problems, and prosocial behavior. An internalizing score was computed as a sum of emotion problems and peer relationship problems, an externalizing score was summed with the conduct and hyperactivity subscales, and a total difficulty score was generated by combining these four subscales. (Internalizing: α_*pre*_ = 0.75; α_*post*_ = 0.81; α_*follow-up*_ = 0.85; Externalizing: α_*pre*_ = 0.83; α_*post*_ = 0.85; α_*follow-up*_ = 0.90; Global: α_*pre*_ = 0.85; α_*post*_ = 0.88; α_*follow-up*_ = 0.92).

## Data Availability

Data is not publicly available now due to ongoing secondary analyses. If researchers wish to analyze data jointly, please contact the corresponding author.
